# Obesity-induced inflammation: The impact of the hematopoietic stem cell niche

**DOI:** 10.1172/jci.insight.145295

**Published:** 2021-02-08

**Authors:** Emily Bowers, Kanakadurga Singer

**Affiliations:** Department of Pediatrics and Department of Molecular and Integrative Physiology, University of Michigan Medical School, Ann Arbor, Michigan, USA.

## Abstract

Obesity and obesity-related diseases like type 2 diabetes (T2D) are prominent global health issues; therefore, there is a need to better understand the mechanisms underlying these conditions. The onset of obesity is characterized by accumulation of proinflammatory cells, including Ly6c^hi^ monocytes (which differentiate into proinflammatory macrophages) and neutrophils, in metabolic tissues. This shift toward chronic, low-grade inflammation is an obese-state hallmark and highly linked to metabolic disorders and other obesity comorbidities. The mechanisms that induce and maintain increased inflammatory myelopoiesis are of great interest, with a recent focus on how obesity affects more primitive hematopoietic cells. The hematopoietic system is constantly replenished by proper regulation of hematopoietic stem and progenitor (HSPC) pools in the BM. While early research suggests that chronic obesity promotes expansion of myeloid-skewed HSPCs, the involvement of the hematopoietic stem cell (HSC) niche in regulating obesity-induced myelopoiesis remains undefined. In this review, we explore the role of the multicellular HSC niche in hematopoiesis and inflammation, and the potential contribution of this niche to the hematopoietic response to obesity. This review further aims to summarize the potential HSC niche involvement as a target of obesity-induced inflammation and a driver of obesity-induced myelopoiesis.

## Introduction

The global prevalence of obesity has risen dramatically over the past decades, leading to an increased incidence of obesity-related diseases — including type 2 diabetes (T2D), cardiovascular disease, liver disease, renal failure, and cancer — and increased mortality (1–5). The prevalence of obesity has generated a strong need to better understand mechanisms that lead to obesity-induced disease. While chronic, low-grade inflammation (called metainflammation) is a known contributor to obesity-related diseases (1–7), the switch toward proinflammatory myeloid cell production is not well understood and could provide insights into therapeutic targets.

Adipose tissue (AT) expands in response to excess lipids, primarily for nutrient storage, and has a robust immune component. During steady-state, AT maintains metabolic homeostasis through communication between adipocytes and tissue-resident immune cells, the most predominant being AT macrophages (ATMs) (8). The inflammatory state of ATMs fluctuates depending on nutritional status (9–13). Acute AT inflammation following a high-fat meal is believed to contribute to AT remodeling for storage of additional lipids (14); however, during obesity, adipocytes expand, and signals drive a sustained inflammation in the AT, leading to generation of persistent inflammatory stimuli that originate from excessive adipocyte hypertrophy and further recruit monocytes to AT, thus exacerbating inflammation. This sustained inflammatory loop ultimately promotes cellular and systemic insulin resistance, as inflammatory cells and signals permeate into other tissues, including liver, pancreas, muscle, and brain (1–2, 15–17).

**Figure 1:**
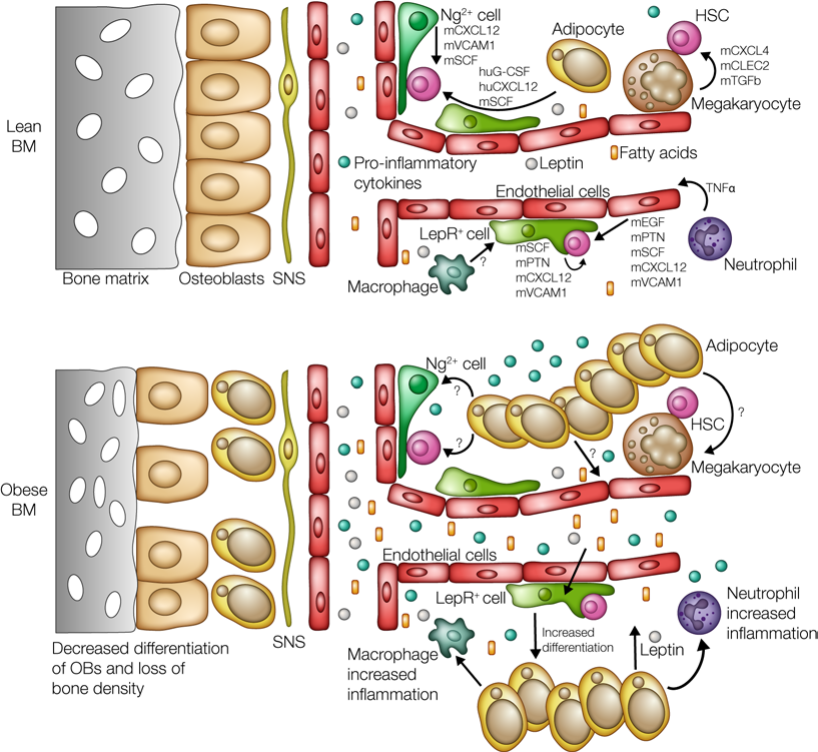
Obesity-induced alterations of the BM and HSC niche influence hematopoiesis. Hematopoiesis is tightly regulated by cytokines produced through the HSC niche, and research has begun to identify the individual contribution of each of these populations in the regulation and maintenance of the HSC pool (top panel). During obesity (bottom panel), the BM landscape changes dramatically. Increased production of leptin from expanding adipose tissue leads to biased differentiation of LepR^+^ perivascular cells toward adipogenesis at the expense of osteogenesis, leading to a significant loss of bone density. While the impact of this physical expansion of the BMAT on the rest of the HSC niche and hematopoiesis is not yet known, this increase would bring many more hematopoietic and stromal cells into direct and/or closer contact with BMAT. Additionally, the expanding AT leads to increased production of proinflammatory monocytes, macrophages, and neutrophils, which in turn leads to an increase in the basal levels of inflammatory cytokines. Chronic exposure to HFD also leads to increased levels of circulating free fatty acids, which could act on both stromal and hematopoietic cells to drive and sustain production of inflammatory myelopoiesis. Illustrated by Rachel Davidowitz.

Expansion and accumulation of ATMs is partially due to proliferation (18–19), but it is also due to increases in circulating proinflammatory monocytes (7, 20, 21), a finding noted in both obese patients (CD14^+^ monocytes) and mice (Ly6c^hi^ monocytes). Circulating monocytes are produced in the BM and then migrate into tissues, via the MCP-1/CCR2 chemokine axis, where they further differentiate into inflammatory macrophages (1, 22). Circulating activated neutrophil populations also increase and migrate into and out of expanding AT, where they promote tissue inflammation and metabolic dysfunction (23–26). While the role of leukocytes in the promotion and propagation of inflammation in obesity-related dysfunctions continues to be covered, the mechanism driving myeloid progenitor expansion is not well understood. Here, we will discuss what is known about the factors propagating inflammation intrinsic to hematopoietic cells but also within the hematopoietic BM niche (Figure 1). Several gaps currently exist, providing an opportunity to further elucidate the mechanism of metainflammation.

## Obesity and hematopoietic stem cells

### Hematopoietic stem and progenitor cell pool fluctuations during obesity.

Hematopoiesis is a tightly regulated process that allows for the continued production of the entire hematopoietic system. This process is highly dynamic and responds accordingly when exposed to different environmental stressors before returning to homeostatic hematopoiesis. As the BM is the primary site of active hematopoiesis and home to hematopoietic stem and progenitor cells (HSPCs), BM populations during obesity and chronic inflammation have been a focus of investigation (7, 27–30). Similar to AT and peripheral blood, BM exhibits induced expansion of monocytes and neutrophils in response to obesity. Interestingly, this quantitative expansion also extends to more primitive hematopoietic cells (7, 27–30). Multiple groups have shown obesity-induced increases of multiple early myeloid progenitor populations, including multipotent progenitors (MPPs), common myeloid progenitors (CMPs), pregranulocyte and macrophage progenitors (Pre-GMs), and granulocyte and macrophage progenitors (GMPs) (7, 27–30). Moreover, an increase in circulating CD34^+^ hematopoietic progenitors has been reported in obese patients (31).

Hematopoietic stem cells (HSCs) in obesity remain controversial, as labs have identified expansion (7, 27), reduction (28, 30), and no overall change (29) in HSC pool size in obese mouse models. BM transplant (BMT) studies conducted to functionally test the repopulating ability of the HSC pool in obesity have also produced conflicting data. Some groups have observed an enhanced production of inflammatory myeloid cells from high-fat diet–derived (HFD-derived) BM in both competitive and serial transplantation assays (7, 27), while others have identified impairments in HSC repopulation potential linked to altered quiescence, self-renewal, and impaired differentiation (28–30). However, a further look at these studies indicates that variability in experimental design contributes to these differences. These variations include use of genetic models of obesity (e.g., *ob/ob* and *db/db* mice) versus diet-induced obesity, models of which may also differ in the percent of calories from fat, length of diet exposure, and BMT design. It should also be noted that leptin, whose signaling is modified in *ob/ob* and *db/db* strains and elevated in obesity, has been researched as a possible regulator of the hematopoietic system. Studies going back to the 1990s have implicated leptin signaling as a positive regulator of HSC proliferation and differentiation (32–34), thus raising the question of the developmental impacts on hematopoiesis prior to the onset of obesity in an environment devoid of functional leptin or leptin receptors, as in the *ob/ob* and *db/db* mice, respectively.

Obesity, regardless of model, is marked by increased levels of a wide array of proinflammatory cytokines, like IL-6, TNF-α, and IL-1β, in the expanding tissues and circulation. These proinflammatory signals further promote inflammation through signaling pathways like TLR4 (35–38). While there is consensus that inflammation is a major contributor of the obesity phenotype, the exact mechanisms driving increased myelopoiesis in obese models is unclear, with both dietary components and inflammatory pathways implicated. The impact of inflammation itself on the hematopoietic system has been studied for decades, but the role of metainflammation on HSPC populations has only recently been explored (39–42). HSCs remain predominantly quiescent during steady state, with more committed downstream progenitors taking on the majority of the hematopoietic production (43). However, in response to inflammatory stressors, the HSC compartment becomes active, leading to increased proliferation and altered production of mature blood cells (44). Inflammatory signals, like IL-1β, TNF-α, IL-6, and TLR ligands, have all been shown to impact the HSC pool during acute and chronic inflammation and, interestingly, are also all elevated during obesity.

### Inflammatory cytokines and hematopoiesis.

Early research using in vitro cultures of primitive HSPCs, including human CD34^+^ cells and murine Lineage^–^Sca-1^+^cKit^+^ (LSK) cells, suggested a role for proinflammatory cytokines, like IL-1β, TNF-α, and TLR ligands, in the induction of hematopoiesis (45–47). In vitro studies have implicated IL-1β (48), TNF-α (49–52), and TLR (53–57) signaling in accelerated proliferation and myeloid differentiation of human and murine HSPCs. This enhanced HSPC proliferation and differentiation has been linked to increased expression of cell cycle activators (cyclin C, cyclin E1, cyclin-dependent kinase 4, and *Myc*) and myeloid lineage genes (GM-CSF receptor, PU.1, GATA-1, and C/EBPα), along with reduced expression of proliferation inhibitors like p57 (48, 55). TNF-α signaling has also been linked to increased HSC repopulation potential following BMT (52). While elevated IL-6 levels promote myelopoiesis and inhibit lymphopoiesis, its role during an inflammatory state is not well studied (58, 59). However, in vitro cultures and BMT studies from other groups have suggested that TNF-α inhibits HSPC function due to increased apoptosis and impaired self-renewal (49, 51, 60, 61). Recent work has suggested that TNF-α effects may be concentration dependent, as increasing TNF-α levels proportionally increases negative effects on HSCs (62). This dosage effect varies between different HSPC populations, as GMPs respond differently than HSCs. These data suggest that HSPC populations are differentially regulated by not just the presence or absence of TNF-α, but also higher or lower TNF-α (and possibly other cytokines), within the environment.

### Mechanisms of direct obesity-induced hematopoiesis.

While many gaps remain in the study of inflammation in hematopoiesis, current literature suggests that activation of inflammatory pathways in HSPCs significantly influences their function, specifically in decisions concerning proliferation versus quiescence and differentiation versus self-renewal. There is also a large overlap in hematopoietic phenotypes, including enhanced myelopoiesis and HSPC expansion, observed in obesity and inflammation. Genetic loss of TLR4 signaling (via deletion of *Tlr4* or downstream regulators *Myd88* or *Ticam1*) in obese mice through whole-body KOs (36, 37) and BM chimeras (35, 38) leads to decreased levels of TNF-α and IL-6 (35), improved insulin sensitivity (35, 38), decreased activation of proinflammatory ATMs (36, 38), and decreased AT fibrosis (37). Loss of TLR4 signaling also alters the HSPC phenotype in the BM of obese mice. *Tlr4^–/–^*, *Myd88^–/–^,* and *Ticam1^–/–^* mice placed on HFD for 16 weeks had decreases in GMPs and decreased production of myeloid colonies via CFU assay (38). Hematopoietic-specific deletion of *Tlr4* has been achieved with chimeric transplant systems generating recipient mice that contain BM either entirely (7, 28, 32) or partially (27, 28, 38) devoid of TLR4 in hematopoietic cells. Exposure of these mice to HFD decreased HSC, CMP, and GMP populations compared with the WT HFD controls. HSCs isolated from TLR4-deficient HFD-fed mice for BMT showed normal reconstitution compared with the myeloid-biased reconstitution noted from WT HFD HSCs.

Other labs have also investigated the involvement of IL-1β and the NLRP3 inflammasome in the propagation of myelopoiesis in obesity (7, 39). *Il1r^–/–^* mice placed on HFD for 6 months have less expansion of HSCs, CMPs, and GMPs compared with the *Il1r^+/+^* HFD controls and decreased production of inflammatory cytokines from BM-derived macrophages (7). Reciprocal transplants of WT or *Nlrp3^–/–^* BM into irradiated *ob/ob* recipients also suggest a role for the inflammasome in the obesity-induced BM phenotype. Five weeks after transplant, recipient mice that received *Nlrp3^–/–^* BM had less expansion of GMPs, and circulating monocytes and neutrophils, compared with WT BM recipients (7).

Ki67 staining has shown increased cellular proliferation of the HSC, MPP, CMP, and GMP populations of obese mice (7, 28, 63). Obese HSPCs also exhibit consistent increases in expression of genes associated with myeloid differentiation (*Csfr1, Spi1*, and *Runx1*), myeloid activity (*Stat3* and *Stat6*), and cell cycle activators (*Cdk1* and *Ccna2*) (28). Increased *cMyc* expression, which promotes the cell cycle, and decreased expression of cell cycle inhibitors *p21* and *p57* have also been reported in purified obese HSCs. This gene expression profile is believed to be the result of lipid raft loss on the HSC cellular membrane, resulting in inhibited TGF-β signaling, which has been shown to nudge HSCs out of quiescence and into proliferation (63). However, other groups have identified increased quiescence at the expense of differentiation and impaired long-term reconstitution capability in HSCs from mice exposed to chronic HFD. Increased quiescence is associated with increased *Gfi1* and *Cdkn1* expression of HSCs isolated from obese animals (29). The majority of these studies used different models, which could heavily impact the results. However, these studies shed light on the complexity of obesity and inflammation, and the dynamic changes that can occur within the hematopoietic system in response to the environment (both locally and systemically).

## The role of the HSC niche in hematopoiesis

The HSC niche is a multicellular structure within the BM composed of nonhematopoietic stromal cells (adipocytes, osteoblasts [OBs], endothelial cells, perivascular mesenchymal stem cells [MSCs], and sympathetic nerves) and hematopoietic cells (megakaryocytes [Mks], macrophages, and neutrophils) that provide instructional signaling to associated HSCs. HSC niche–derived signals are important in the maintenance and proper functioning of the HSC pool during steady-state and stressed conditions, as these signals balance HSC proliferation, quiescence, differentiation, self-renewal, extravasation into circulation, and homing into the BM (64–68). While emphasis has been placed on interactions between the niche and HSCs, there is growing evidence that HSPCs residing within BM niches may also rely on these signaling pathways for proper regulation and function (65). As these populations are critical for hematopoiesis, understanding how obesity-driven inflammation impacts niche population function and the contributions of the niche to obesity-associated hematopoiesis alterations is important.

### The role of BM adipocytes in hematopoiesis.

Bone marrow AT (BMAT) has been an enigma in the hematopoietic and HSC niche fields. BMAT is heterogenous and can be divided into 2 populations: constitutive BMAT (cBMAT), which is densely packed and is present from birth, and regulated BMAT (rBMAT), which is more dispersed within the BM and capable of both expansion and contraction throughout life (6, 69). Studies initially linked BM adiposity with inhibited hematopoiesis based on observations that bones containing higher amounts of BMAT had less active hematopoiesis (70, 71). However, newer data point to a possible positive regulatory role for BMAT on hematopoiesis. Coculture of adipocytes and primary hematopoietic cells supports increased HSPC differentiation into mature blood cells (72, 73), and recent work suggests that BMAT is a source of hematopoietic and HSC regulatory cytokines. A study of human BMAT implicated this niche as a source of CXCL12 and G-CSF (74), while studies of murine BMAT suggest it is a source of SCF during hematopoietic regeneration following lethal irradiation and transplantation (75). The specific role of BMAT in the regulation of hematopoiesis is still under investigation; however, adipocyte-derived adipokines have been shown to regulate HSCs (6, 32-34, 69, 74, 76–78). Adipose-derived adiponectin promotes HSC proliferation and BMT, reconstituting potential through p38 MAPK activation (77) and mTORC signaling (78). Leptin, another adipokine, has also been shown to promote HSC expansion and differentiation (32–34), though the intracellular mechanism inducing proliferation is not known. During obesity, circulating leptin increases while adiponectin decreases. While both adipokines have been shown to promote HSC proliferation, their specific role during obesity is not known. However, expanding BMAT could provide a local source of leptin to promote HSC expansion. While scarce, some data suggest that AT-derived fatty acids could have regulatory roles in myelopoiesis (79, 80) and HSC pool maintenance (81). Additionally, prostaglandin E_2_ (PGE2), which is also elevated during obesity and derived from the fatty acid arachidonic acid, has been shown to inhibit B lymphopoiesis, suggesting another possible mechanism for increased myelopoiesis or skewing in obese BM (82).

As expanding white AT (WAT) increases production of inflammatory cytokines and receptors (83–85), BMAT also increases production of inflammatory cytokines like IL-6, TNF-α, and serum amyloid A3 (SAA3) (76, 86–88). In humans, BMAT expresses higher basal levels of IL-6 and TNF-α compared with WAT (87). Interestingly, recent data have suggested that a switch occurs during obesity wherein secretion of these factors is significantly lower in BMAT compared with expanding WAT (89). This discovery emphasizes the complexity and heterogeneity of the different AT depots, their response to obesity, and their impact on hematopoietic system regulation.

BMAT is considered a hybrid of white and brown adipocytes; however, during HFD, BMAT appears to undergo a shift toward a more WAT-like phenotype (90). Comparison of BMAT and WAT during homeostasis identified differential lipid metabolism, with decreased lipolytic activity and a shift toward cholesterol-oriented metabolism in BMAT compared with WAT (91). However, how BMAT lipid metabolism is impacted during obesity is still unknown. As WAT is typically associated with energy storage and BAT with energy expenditure, the observed metabolic shift in BMAT during HFD-induced obesity could be the result of an increased lipid storage demand. Additionally, it is not clear how this shift in metabolic signature in BMAT impacts hematopoiesis. Extensive research has gone into the identification and characterization of WAT and BAT depots during both homeostasis and metabolic dysfunction, while much less is known about the metabolic contributions of the BMAT — let alone the alterations that occur in this depot during conditions like obesity. This emphasizes the need to better understand the role of BMAT in both systemic metabolism and hematopoiesis.

### The role of endothelial cells in hematopoiesis.

The close proximity of HSCs to the BM vasculature was an initial clue that BM endothelial cells (BMECs) play a role in the regulation of HSCs (92–98). BMECs induce HSC proliferation and differentiation through activation of AKT (94) and the Notch signaling pathway (95, 96), while in vitro MAPK activation in BMECs induces significantly less expansion of hematopoietic cells (94), suggesting that various activation states within the BMEC population could have differing effects on the associated HSCs. BMECs are a major source of SCF, CXCL12 (93, 98–100), BMEC-derived EGF, and pleiotrophin (101–103), which are critical for homeostasis and hematopoietic system regeneration following BMT (97). BMECs make up a heterogenous population composed of arteriolar ECs (AECs) and sinusoidal ECs (SECs). While differences in production of all known HSC regulatory genes are not fully elucidated, AEC-derived SCF has been observed to be more critical for HSCs than SEC-derived SCF (99). HSCs also exist in different proliferative states depending on the BMEC niche. HSCs associated with AECs are found in a more quiescent state than those associated with SECs (104, 105); however, whether this is strictly due to the BMECs themselves or influenced by additional niche populations is not known.

BMECs express a range of TLRs (TLR1–7, TLR9) and inflammatory receptors, such as those for TNF-α, IL-1, and IL-6 (106–108), suggesting a role in stress-induced hematopoiesis. Early in vitro assays of human EC lines identified increased expression of the myeloid differentiation cytokine GM-CSF upon IL-1 or TNF-α stimulation, suggesting that BMECs contribute to demand-dependent hematopoiesis (109, 110). In recent years, it has become apparent that G-CSF–mediated emergency granulopoiesis is heavily dependent on inflammatory signaling through BMECs, in part through TLR4 (111–114). Additionally, LPS and TNF-α induce expression of Jagged-2 on BMECs and Notch-1 and -2 on hematopoietic cells, suggesting that these pathways may work together to promote Notch signaling in the BM during inflammation (115). Chronic activation of the inflammatory stress–associated MAPK pathway in BMECs (116, 117) promotes aberrant NF-κB signaling, which is believed to drive myeloid bias in HSCs ex vivo at the expense of self-renewal (118). While the majority of this research has been within the context of acute infection and the associated immune response, several cytokine and signaling pathways (IL-1β, TNF-α, and TLR4) and the skewing toward myelopoiesis are also implicated in obesity, making these pathways prime targets for further analysis. While not studied during infection, expression of Del-1 by BMECs has also been shown to regulate homeostatic production of myeloid cells, suggesting a possible signaling pathway that is disrupted during obesity (119).

Significant impairments in BMECs of the HSC niche have been observed in the context of diabetes (120–122). While some studies focus on type 1 diabetes (T1D), which addresses the impact of hyperglycemia and differs from the obese conditions associated with T2D, they still provide insight into the effect of metabolic dysfunction on the BM. When mice are exposed to streptozotocin to induce β cell failure, the BM undergoes significant loss of hematopoietic cells, a dramatic decrease in vascular density, and increased vascular permeability (120, 122). BMECs from streptozotocin-treated mice also have increased levels of ROS and decreased migration toward chemokines CXCL12 and VEGF-A (120, 121). BMECs from these mice also exhibit decreased AKT pathway activation, which is associated with impaired BMEC migration, leukocyte extravasation, and vascular permeability (122).

The role of BMECs in metainflammation are limited, but recent work described BMEC-specific EGFR signaling in mediating the BM phenotype observed in obesity (123). The authors noted a significant reduction in CXCL12 in BMECs from obese mice; however, increased HSC localization at the BM vasculature was observed in obese mice compared with controls. These results are conflicting, as CXCL12 is considered the prominent cytokine for HSC retention in the BM, and a reduction in CXCL12 would not typically associate with increased HSC recruitment. BMEC-specific EGFR deletion in *Cdh5-Cre;EGFR^fl/fl^* mice had no effect on CXCL12 expression in response to HFD, and EGFR-deficient mice underwent increased expansion of HSCs, along with notably more myelopoiesis compared with the WT HFD group. The authors of this study conclude that BMEC EGFR signaling is protective and aids in maintenance of normal hematopoiesis. One possible caveat of this study arises from the use of *Cdh5-Cre* mice, as constitutive deletion in BMECs could result in deletion in the hematopoietic system (86). Overall, these data suggest that obesity and hyperglycemia influence the HSC niche by both promoting enhanced myelopoiesis and affecting mechanisms that keep hematopoiesis in check (124).

### The role of perivascular stromal cells in hematopoiesis.

Perivascular stromal cells are found throughout the BM space, although they are most frequently found in close association with BM vasculature (93, 104). Recent work has further characterized this population into, roughly, 2 distinct subtypes: leptin receptor^+^ (LepR^+^) cells (98), which are relevant to obesity, and the Ng2^+^ population (104). Perivascular stromal cells not only show characteristics of MSCs, but also express high levels of critical HSC regulatory proteins, including SCF, CXCL12, VCAM1, and Ptn (67, 98, 104, 125, 126). BM stromal cell–derived TNF-α has also been shown to regulate HSC biasing toward myelopoiesis via activation of angiotensin II (127). Mice lacking the angiotensin II receptor in their BM stroma have decreased production of granulocytes and macrophages and reduced GMPs (127), pointing to an involvement of the BM niche in promoting HSC skewing. Recent data have also suggested that these CXCL12 sources are important for maintenance of the MPP population (128). Thus, it is possible that the niche is not specific to HSCs but regulates hematopoiesis through multiple stages of differentiation.

BM MSCs have been implicated in regulating both the innate and adaptive immune response through the production of cytokines and inflammatory factors, such as TNF-α, IL-10, IL-6, PGE-2, NO, and CXCL12 (129, 130). The effect of these cells on the immune system is quite varied, as BM MSCs can both inhibit (131–135) and promote (136–140) immune responses. MSCs can inhibit T cell responses (via IFN-γ, TNF-α, or IL-1β) (141–143) and promote antiinflammatory signaling in macrophages (via PGE-2 and TLR2) (134, 135). Activation of MSC TLRs promotes the increased production and secretion of proinflammatory cytokines (IL-1β, IL-6, TNF-α, CCL5, and type 1 IFNs; refs. 136, 137), which could increase inflammation in the BM environment. MSC-derived IL-6 promotes expansion of myeloid progenitors and mature myeloid cells (138, 139), and TLR4 signaling on MSCs directly leads to secretion of the monocyte chemokine MCP-1 (also known as CCL2) to promote the extravasation of inflammatory Ly6c^hi^ monocytes into circulation (140), another mechanism that could be exacerbated during obesity.

We have a limited understanding of the role of perivascular MSCs during obesity-induced hematopoiesis. In T1D, BM MSCs become nonresponsive to G-CSF signaling, leading to a maintained CXCL12 expression and impaired mobilization of HSPCs (144). BM MSCs are capable of differentiating down the osteogenic or adipogenic pathways, and data taken from obese mice heavily suggest that differentiation is altered during obesity. LepR^+^ perivascular cells are targeted by elevated leptin and differentiate into adipocytes at the expense of OBs, leading to BMAT accumulation and decreased bone density (6, 145). However, it is not clear if differentiation of these MSCs would lead to HSC niche loss or if this would contribute to obesity-associated hematopoietic phenotypes.

### The role of the sympathetic nervous system (SNS) in hematopoiesis.

The SNS innervates the BM by traveling along the arteriolar vasculature (146). The SNS helps prevent HSC exhaustion through production of TGF-β, which pushes HSCs into a quiescent state (146). Catecholamines produced by the SNS can directly act on HSCs or aid in the BM response to G-CSF–mediated mobilization by altering CXCL12 expression (147–151). Treatment of human CD34^+^ HSPCs with a dopamine agonist in the presence of G-CSF prior to BMT increased hematopoietic colonies and increased engraftment into NOD-SCID recipient mice (150).

While the use of adrenergic receptor agonists on both human and murine immune cells leads to decreased production of cytokines (TNF-α, IL-6, IL-12, and IFN-γ) and increased migration (149, 152), there is limited information on how SNS signals affect either BM immune cells or HSPCs during inflammatory stress and obesity. Some data suggest that T1D impairs SNS-induced mobilization of HSCs following G-CSF treatment by preventing CXCL12 downregulation in BM MSCs (144). A recent model of SNS-driven atherosclerosis (a comorbidity of obesity) led to a reduction in HSC niches — like sinusoidal endothelial cells — and extravasation of HSPCs out of the BM and into the spleen, suggesting severe BM dysfunction (153). This correlated with increased production of neutrophil-derived proteases capable of targeting a critical HSPC chemokine receptor, CXCR4 (153). Treatment of these mice with an adrenergic receptor blocker prevented CXCR4 cleavage, suggesting a possible mechanism by which SNS targets neutrophils to regulate HSPC trafficking into and out of the BM. However, the impact of chronic low-grade inflammation and obesity on the SNS and its role in regulating hematopoiesis is still largely unknown and clinically important to investigate.

### The role of OBs in hematopoiesis.

OBs are responsible for bone synthesis and mineralization, and they are capable of positively regulating hematopoiesis and HSC function (154, 155). In vivo murine models have suggested that OBs can modulate the size of the HSC pool through increased expression of Jagged-1 (156) and activation of BMP signaling (157). OB-derived angiogenin — another factor elevated in obesity — can promote HSC quiescence, as loss of angiogenin from osteolineage BM cells leads to increased proliferation of the HSC pool (158). However, recent studies have contradicted these data and suggest OB signaling is dispensable for proper HSC maintenance and function (159). Interestingly, CXCL12 deletion from OBs has no effect on the HSC pool but does lead to depletion of early lymphoid progenitors (93, 98), further supporting the existence of niches for hematopoietic progenitors. While the data might still be unclear as to the specific role of OBs in regulation of hematopoiesis, plenty of data support their role during inflammation. Following bacterial infection, OBs are a source of several inflammatory factors (e.g., IL-6, IL-8, CXCL10, and MCP-1) (160–163). Additionally, OBs express several TLRs (TLR2, TLR4, TLR5) and increase IL-6 expression when cultured in the presence of TNF-α or IL-1β, suggesting an active contribution to the immune response (161, 163–165).

Obese patients and mice undergo significant bone loss. Studies suggest that obesity-induced bone loss is due to skewed differentiation of LepR^+^ cells toward adipogenesis instead of osteogenesis (145); however, several cytokines produced both by AT and immune cells can negatively impact maintenance of the OB population and bone remodeling. Leptin and adiponectin have also been implicated as regulators of bone homeostasis. Leptin has been shown to positively and negatively influence the OB pool size. Early in life, leptin is believed to stimulate bone growth; however, later in development and during obesity, it may promote bone loss through induction of RANKL expression, thereby promoting osteoclast production and bone reabsorption (166). Adiponectin is significantly reduced in obesity and appears to have a more protective role on OBs. In vitro and in vivo assays have suggested that adiponectin promotes OB production and inhibits osteoclast formation (167). Moreover, IL-6 and TNF-α, which are elevated during obesity, have been shown to promote osteoclast differentiation (168–170), thereby prompting bone loss. While OBs may not directly impact the HSC pool, loss of this population could negatively impact the production of lymphoid cells in obese mice and humans.

### The role of Mks in hematopoiesis.

Mks are large, multinucleated cells that reside in close proximity to the vasculature, where they shed platelets into circulation. As with other identified niche populations, Mks became of interest due to their close association with HSCs (171–173). Data suggest that Mks induce HSC quiescence through CXCL4, CLEC2, and TGF-β signaling (171–173). More recently, coculture of Mk-derived microparticles with human CD34^+^ HSPCs appears to induce Mk lineage differentiation (174). This work on Mks was among the first to implicate a mature hematopoietic cell as a component of the HSC niche.

Mks are targeted in response to inflammation (175–177). In mice, IL-1β administration increases not only the size of Mks, but also circulating platelets and granulocytes (178, 179). IL-6 has also been shown to influence the output of platelets (179, 180) through increasing levels of thrombopoietin (TPO), a critical cytokine for Mk differentiation and maturation (178, 179). Mks and platelets also express TLRs (TLR1–4, TL6, and TLR9), and treatment with LPS in humans leads to an eventual increase in TPO and platelets (181–186). Platelets may produce additional inflammatory cytokines, and levels of these transcripts have been directly correlated to BMI. Humans with a higher BMI have elevated transcripts encoding IL-1R1, IL-6, TNF-α, TLR2, and TLR4 (187). While platelets have been linked to inflammatory states in systemic lupus (188) and inflammatory arthritis (189), the role of platelets and Mks in the HSC niche, metainflammation, and the increased myelopoiesis seen in obesity still remains unclear.

### The role of macrophages and neutrophils in hematopoiesis.

Surprisingly, Mks are not the only mature hematopoietic population with HSC regulatory function. BM macrophages, and more recently neutrophils, have been implicated as indirect regulators of the HSC pool. Macrophages regulate CXCL12 expression in BM perivascular cells, which may facilitate G-CSF–induced mobilization of HSPCs into circulation (190–192). Additionally, radio-resistant BM macrophages locate in perivascular niches following myeloablation, where they produce PGE2 to promote HSC reconstitution and induce CXCL12 expression in BM stromal cells (193, 194). The clearance of neutrophils by BM macrophages has also been linked to altered expression of CXCL12 in the BM and extravasation of HSPCs (195). Neutrophil-derived TNF-α promotes regeneration of damaged BM vasculature following myeloablation, leading to increased hematopoietic reconstitution (196). While these data suggest that BM macrophages and neutrophils indirectly regulate HSCs, some data indicate that these mature immune cells directly regulate the HSC pool through histamine production (197). These studies suggest that direct mechanisms of HSC regulation by myeloid cells in hematopoiesis have yet to be identified.

BM myeloid cells are important niche components that regulate hematopoiesis. Obesity-induced inflammation is well known to induce expansion of proinflammatory monocytes and neutrophils; however, the impact of this expansion specifically in the BM has yet to be determined. Tissue-resident macrophages, and infiltrating monocytes and neutrophils, are major drivers of inflammation through production of cytokines; however, whether or not BM myeloid cells produce similar levels is not known. Therefore, these cells may promote the transition into an inflammatory BM environment through similar mechanisms. These signals could target both HSPCs and the BM niche to promote production of more inflammatory myeloid cells. While studies suggest defective acute inflammatory responses, they also raise the question as to how functional impairments of these mature myeloid cells could alter HSC regulation in the BM.

## Discussion

Metainflammation is a driver for lifetime mortality and morbidity; hence, it is critical to understand the mechanisms contributing to alterations of hematopoiesis. While the majority of studies conducted have focused on the changes occurring within metabolic tissues such as the AT, the impact of obesity on hematopoiesis is still a new area of research. Several groups have begun identifying alterations to hematopoietic production during the onset of obesity in mice. More specifically, obesity promotes increased production of myeloid cells through biased expansion of myeloid-specific progenitors and skewing of the HSC pool toward myelopoiesis. However, there are gaps in understanding the mechanisms underlying these processes and the impact of obesity on hematopoiesis and the HSC pool (Figure 1).

Going forward, it will be important to further determine if there is a dependence on specific dietary factors, such as saturated fats, or if genetic obesity itself can mimic what is observed in dietary obesity. In addition, the age of mice used in studies is another confounding factor; therefore, understanding the immune changes in both young and aged animals is clinically relevant and necessary, as seen with regard to age-related influence of metainflammation observed in recent pandemic infections (198). The use of either HSPC frequency or cell number are also important to consider in study design and data analysis. Given that cellularity itself might significantly shift in the BM space with obesity and HFD exposure, the use of frequency could be misleading. For example, expansion of a population like neutrophils (one of the most abundant blood cells in the BM) could drastically alter BM cellularity, which would significantly affect the frequency of HSCs, even if the total number of HSCs was unchanged. While this review has focused on the phenotypes attributed to obesity, it is critical to mention that all of these studies utilize male mice, as female mice do not present the same inflammatory signatures (199) or metabolic impairment. A very similar phenotype is also observed in humans, suggesting a possible effect of sex hormones. The study of the HSC niche in female BM could provide critical insight into the regulatory roles of these populations in the chronic inflammatory response.

The HSC niche in obesity has yet to be heavily interrogated. While some studies suggest that obesity impairs BMEC function, increases BM adiposity, and decreases bone density, the impact of these changes on the ability of the niche to properly regulate hematopoiesis remains to be determined. Recent work demonstrates that adipocytes and ECs can communicate through extracellular vesicles (200). Such communication would have large implications into the role of BMAT as a signaling hub in the BM space that could influence hematopoietic decisions.

Given that inflammation is so heavily linked to the pathogenesis of obesity, understanding the cell types within the hematopoietic niche that support or impair hematopoiesis is critical. Focused research into the signals and the role of inflammation in the regulation of hematopoiesis through the HSC niche is essential to understand the therapeutic potential of altering the BM niche to regulate immune responses and improve metabolic health in obesity.

## Version 1 02/08/2021

Electronic publication
